# Molecular phylogeny of 42 species of *Culicoides* (Diptera, Ceratopogonidae) from three continents

**DOI:** 10.1051/parasite/2017020

**Published:** 2017-06-22

**Authors:** Denis Augot, Bruno Mathieu, Leila Hadj-Henni, Véronique Barriel, Sonia Zapata Mena, Sylvia Smolis, Darine Slama, Fano José Randrianambinintsoa, Gabriel Trueba, Matthieu Kaltenbach, Nil Rahola, Jérôme Depaquit

**Affiliations:** 1 USC Vecpar, ANSES-LSA, Université de Reims Champagne-Ardenne, SFR Cap Santé, Faculté de Pharmacie 51 rue Cognacq-Jay EA 4688 Reims 51096 France; 2 Institut de Parasitologie et de Pathologie Tropicale de Strasbourg, Université de Strasbourg, Faculté de Médecine 3 rue Koeberlé EA7292 Strasbourg 67000 France; 3 Muséum National d’Histoire Naturelle, CR2P-UMR 7207 CNRS, MNHN, UPMC 8 rue Buffon CP 38 75005 Paris France; 4 Instituto de Microbiologia, Colegio de Ciencias Biologicas y Ambientales, Universidad San Francisco de Quito, Cumbayá EC170157 Quito Pichincha Ecuador; 5 Laboratory of Medical and Molecular Parasitology-Mycology, Faculty of Pharmacy, University of Monastir 99UR/08-05 5000 Monastir Tunisia; 6 Medical Entomology Unit, Institut Pasteur de Madagascar, Ambatofotsikely BP 1274 Antananarivo 101 Madagascar; 7 Laboratoire de Pharmacologie, Université de Reims Champagne-Ardenne, UFR Pharmacie 51 rue Cognacq-Jay 51100 Reims France; 8 Unité MIVEGEC, UMR 224-5290 IRD-CNRS-UM, Centre IRD de Montpellier BP 64501 911 avenue Agropolis 34394 Montpellier France; 9 Centre International de Recherches Médicales de Franceville (CIRMF) BP 769 Franceville Gabon

**Keywords:** *Culicoides spp.*, Phylogeny, Ecuador, France, Gabon, Madagascar, Tunisia, 28S, COI

## Abstract

The genus *Culicoides* includes vectors of important animal diseases such as bluetongue and Schmallenberg virus (BTV and SBV). This genus includes 1300 species classified in 32 subgenera and 38 unclassified species. However, the phylogenetic relationships between different subgenera of *Culicoides* have never been studied. Phylogenetic analyses of 42 species belonging to 12 subgenera and 8 ungrouped species of genus *Culicoides* from Ecuador, France, Gabon, Madagascar and Tunisia were carried out using two molecular markers (28S rDNA D1 and D2 domains and COI mtDNA). Sequences were subjected to non-probabilistic (maximum parsimony) and probabilistic (Bayesian inference (BI)) approaches. The subgenera *Monoculicoides, Culicoides, Haematomyidium, Hoffmania, Remmia* and *Avaritia* (including the main vectors of bluetongue disease) were monophyletic, whereas the subgenus *Oecacta* was paraphyletic. Our study validates the subgenus *Remmia* (= Schultzei group) as a valid subgenus, outside of the subgenus *Oecacta.* In Europe, *Culicoides obsoletus, Culicoides scoticus* and *Culicoides chiopterus* should be part of the Obsoletus complex whereas *Culicoides dewulfi* should be excluded from this complex*.* Our study suggests that the current *Culicoides* classification needs to be revisited with modern tools.

## Abstract (fr)

Le genre *Culicoides* comprend des vecteurs de maladies animales importantes telles que la fièvre catarrhale et le virus Schmallenberg. Ce genre comprend 1300 espèces classées dans 32 sous-genres et 38 espèces non classées. Cependant, les relations phylogénétiques entre les différents sous-genres de *Culicoides* n’ont jamais été étudiées. Des analyses phylogénétiques de 42 espèces appartenant à 12 sous-genres et 8 espèces non groupées du genre *Culicoides* d’Équateur, de France, du Gabon, de Madagascar et de Tunisie ont été réalisées en utilisant deux marqueurs moléculaires (ADNr 28S domaines D1 et D2 et ADNmt COI). Les séquences ont été soumises à des approches non probabilistes (maximum de parcimonie) et probabilistes (inférence bayésienne). Les sous-genres *Monoculicoides*, *Culicoides*, *Haematomyidium*, *Hoffmania*, *Remmia* et *Avaritia* (y compris les principaux vecteurs de la fièvre catarrhale du mouton) étaient monophylétiques alors que le sous-genre *Oecacta* était paraphylétique. Notre étude valide le sous-genre *Remmia* (= groupe Schultzei) sous la forme d’un sous-genre valide, en dehors du sous-genre *Oecacta*. En Europe, *Culicoides obsoletus*, *Culicoides scoticus* et *Culicoides chiopterus* devraient faire partie du complexe Obsoletus alors que *Culicoides dewulfi* devrait être exclu de ce complexe. Notre étude suggère que la classification actuelle de *Culicoides* doit être revisitée avec des outils modernes.

## Introduction

Biting midges of the genus *Culicoides* Latreille 1809 (Diptera: Ceratopogonidae) are among the world’s smallest haematophagous flies, measuring from 1 to 3 mm, and are described worldwide, except in Antarctica and New Zealand [45]. They are mainly known as vectors of bluetongue virus (BTV), Schmallenberg virus (SBV) and Oropouche virus (OROV) [12].

Currently, approximately 1300 living and 42 fossil species of *Culicoides* have been described worldwide. Their classification includes 32 subgenera [9] and 38 groups although 13% of occurring species remain ungrouped [11]. This classification is exclusively typological, based on common morphological similarities (e.g. characteristics of reproductive organs, wings, antennae and palps), without any phylogenetic considerations. As most species feature spotted wings, the accurate identification of adults is largely based on subtle variations in size, shape and position of spots that form wing patterns [61–63].

In Africa, Asia and Europe, *Culicoides imicola* and the Obsoletus complex (both from the subgenus *Avaritia* Fox) are considered the most important vectors of BTV, SBV and epizootic haemorrhagic disease virus [20, 35, 45, 60]. Other groups of *Culicoides* are also involved in the transmission of these viruses, such as the Schultzei group (now in the subgenera *Remmia* Glukhova and sometimes synonymised with *Oecacta* Poey) [4, 11], *Culicoides pulicaris* and *C. punctatus* (*Culicoides* Latreille), *C. circumscriptus* (*Beltranmyia* Vargas) [45], and *C. paraensis* (*Haematomydium* Goeldi) only for OROV in South America [12].

Since the recent European bluetongue epizootic outbreak, there has been growing interest in DNA barcoding of *Culicoides* based on the mitochondrial DNA (mtDNA) cytochrome oxidase I (COI) gene, ribosomal (rDNA) regions such as internal transcribed spacer 1 (ITS1) and internal transcribed spacer 2 (ITS2), and the nuclear CAD gene [29]. The rise of DNA barcoding and the lack of taxonomic experts thus enabled COI sequencing to become a tool for rapid identification of *Culicoides* species [1].

Ribosomal DNA markers have been used to investigate phylogenies of closely related species (ITS1 and ITS2: [25, 26, 49]; 28S: [27, 28]), interspecific genetic distances (ITS1 [43, 47]) and population structure (ITS1: [53]) within *Culicoides*. The sequences obtained with ITS1 are generally of low quality [47]. Polymerase chain reaction (PCR) products with ITS2 seem to include many different sequences, even from one individual sample [38].

The lack of phylogenetic data about *Culicoides* does not allow hypotheses about the vector competence for diseases caused by different *Culicoides*-borne viruses. Due to the wide distribution and the great economic importance of veterinary diseases transmitted by biting midges, it seems important to build a modern classification of these insects based on phylogenetic studies to help in epidemiological analyses.

In this study, we carried out a phylogenetic analysis of 42 *Culicoides* species from Europe, America and Africa (including Madagascar) using specimens available in our laboratory. Our sampling included major proven vectors of diseases (i.e. subgenera *Avaritia*, *Culicoides, Haematomydium,* and Schultzei group). In each case, the mtDNA COI and the D1 and D2 regions of the 28S rDNA were analysed. The latter regions were chosen based on the fact that they appear to contain major phylogenetic information at the considered taxonomic level [18, 30, 51] especially for *Culicoides* [27, 28, 58].

## Material and methods

### Collection of *Culicoides* and identification

**Figure 1.**
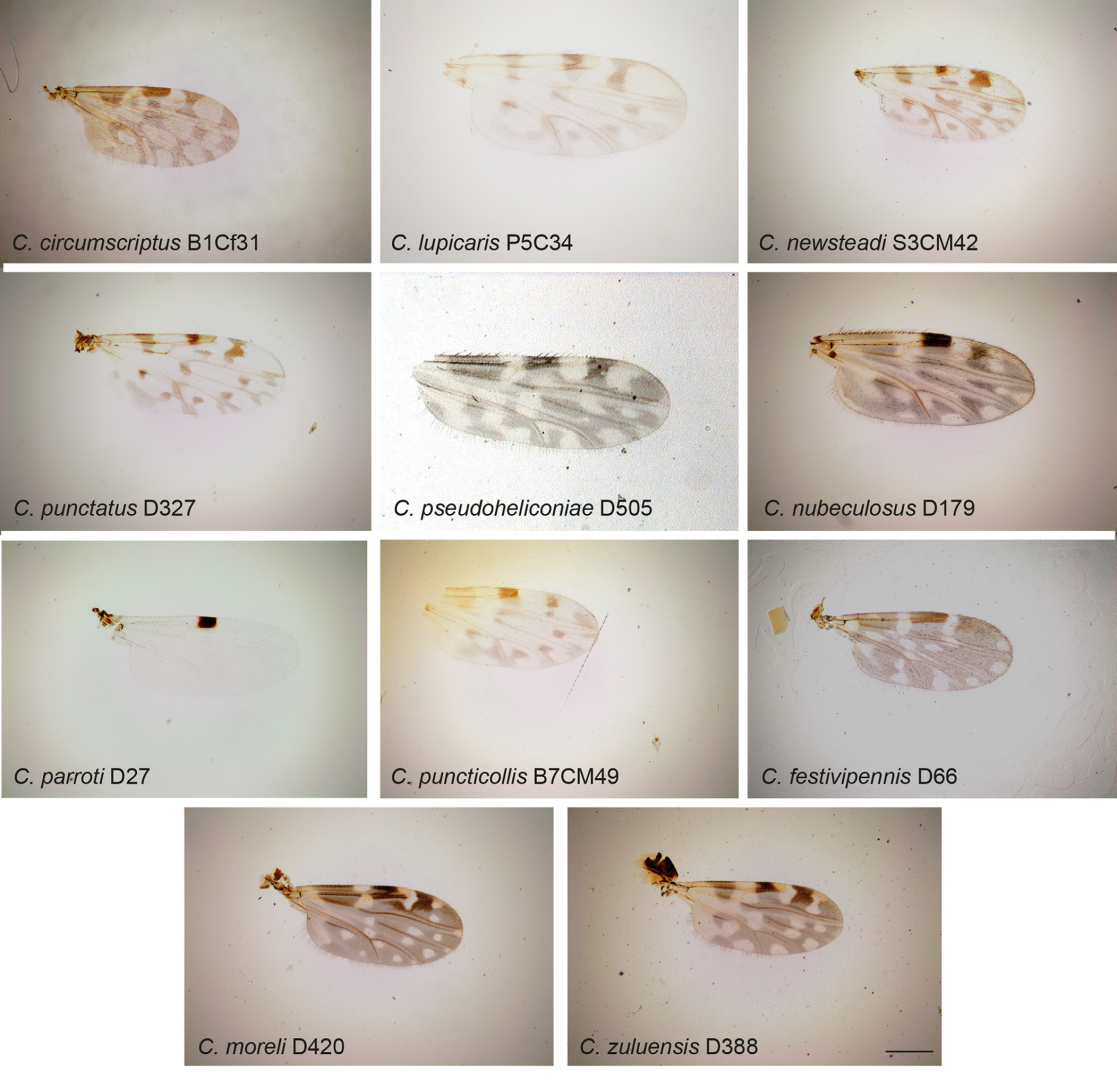
*Culicoides* wing pattern details of species included in our study. The specimen codes are linked with the table. The wings were photographed using a ×4 lens. Bars = 200 μm.

**Figure 2.**
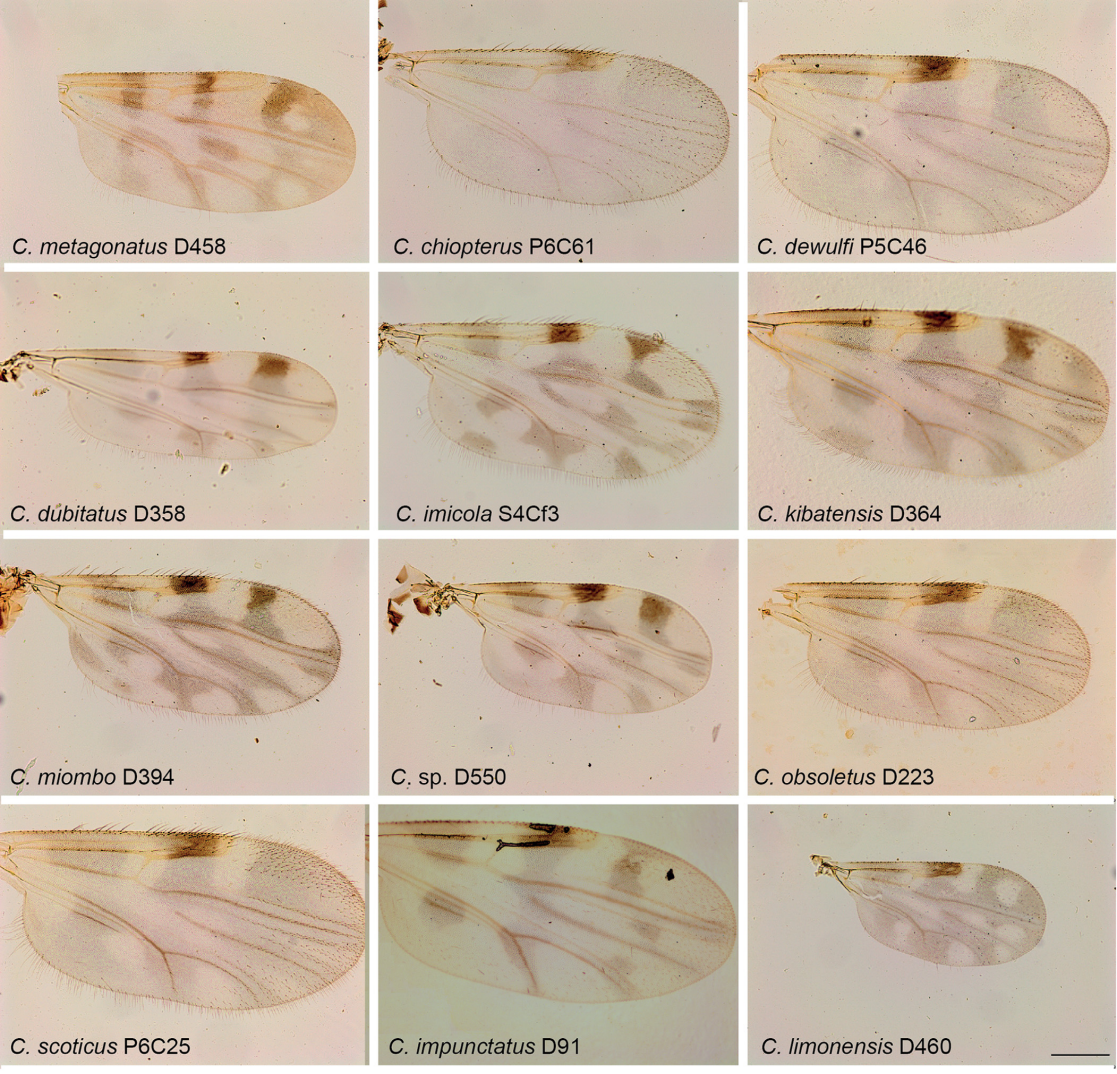
*Culicoides* wing pattern details of species included in our study. The specimen codes are linked with the table. The wings were photographed using a ×10 lens. Bars = 200 μm.

**Figure 3.**
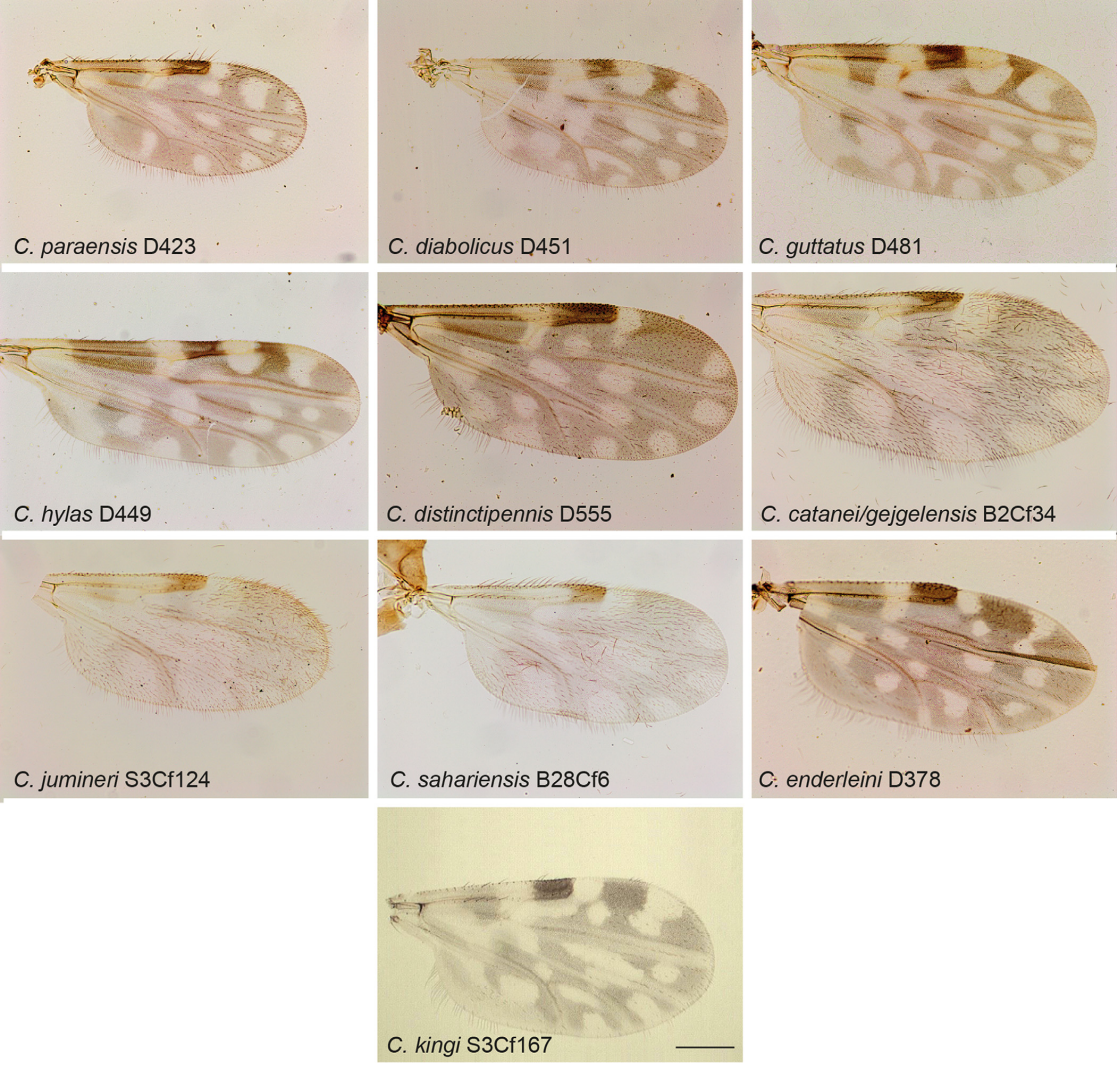
*Culicoides* wing pattern details of species included in our study. The specimen codes are linked with the table. The wings were photographed using a ×10 lens. Bars = 200 μm.

**Figure 4.**
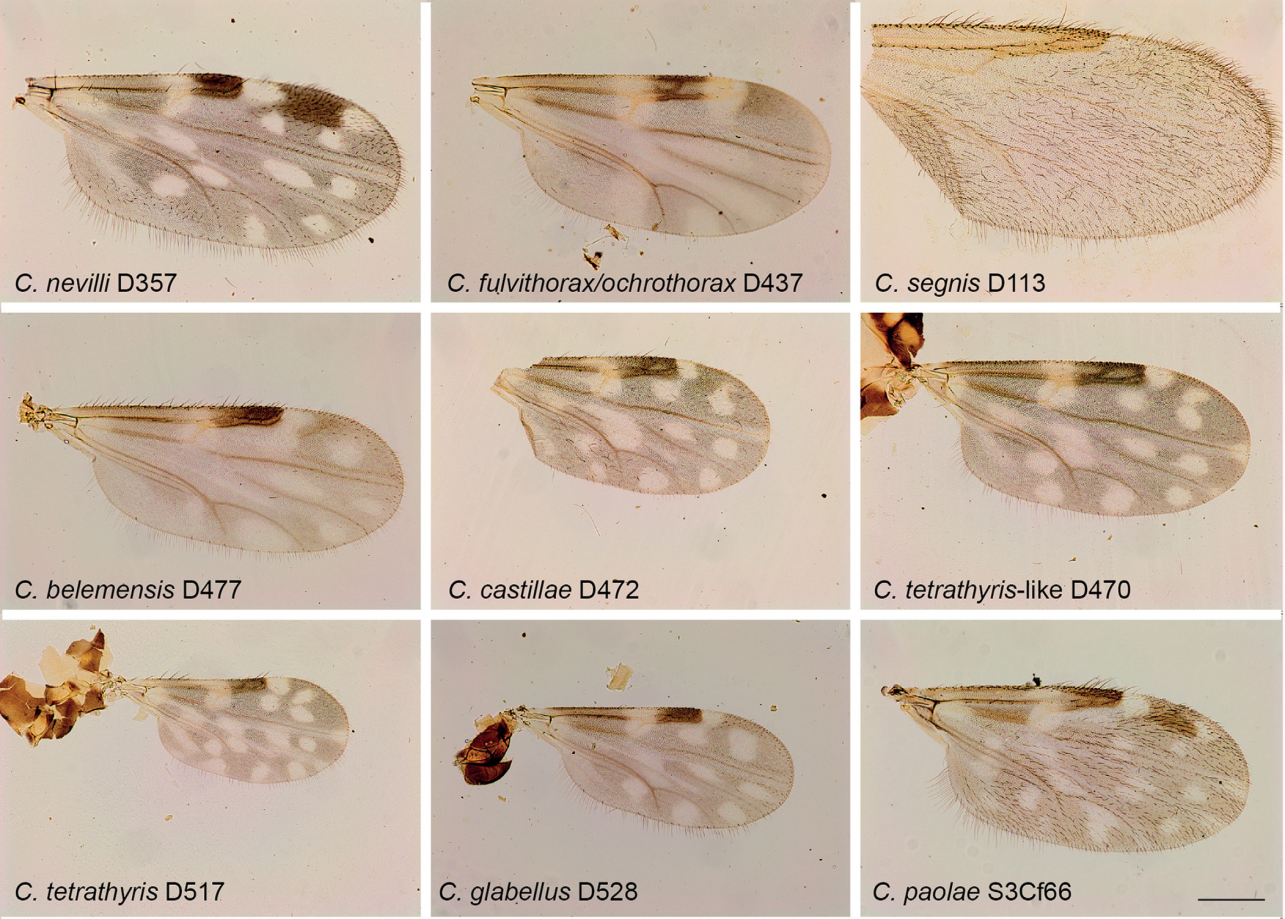
*Culicoides* wing pattern details of species included in our study. The specimen codes are linked with the table. The wings were photographed using a ×10 lens. Bars = 200 μm.

Midges were collected in Ecuador, France, Gabon, Madagascar and Tunisia between 2009 and 2010 using ultraviolet CDC traps and standard CDC miniature light traps (John W. Hock Company, Gainesville, FL, USA). Insects were stored in ethanol 95°. Specimens were identified to species, species group or subgenera (Table 1; Figs. 1–4) using different morphological keys [13, 14, 17, 21, 22, 24, 31, 36, 62].

**Table 1.** List of *Culicoides* spp. used in the phylogenetic analyses, classification of Borkent, 2014.

Subgenus	Taxa present		Country	No. (codification)	GenBank accession number	
	Group	Species			COI	D1D2
*Anilomyia*		*C. metagonatus*	EC	EC-meta-1-D458	KY707782	KF286339
		*C. chiopterus*	FR	FR-chio1-P6C61	KY707805	KF286340
		*C. dewulfi*	FR	FR-dew1-P5C46	HM022877	KF286341
				FR-dew2- P3C17*	HM022878	
		*C. dubitatus*	MA	MA-dub1-D358	KY707796	KF286342
			GA	GA-dub2- D558	KY707795	KF286343
			MA	MA-dub3 D379	KY707797	KF286344
*Avaritia*		*C. imicola*	TU	TU-im1-S4Cf3	KJ729975	KF286345
				TU-im2-S6Cf111*	KJ729976	
		*C. kibatiensis*	MA	MA-kib1-D364*	KY707781	KF286348
				MA-kib2-D401		
		*C. miombo*	MA	MA-mio1-D394*	KY707800	KF286349
				MA-mio2-D412		
		*C. sp.*	GA	GA-img1-D439	KY707791	KF286346
				GA-img2-D550	KY707790	KF286347
		*C. obsoletus*	FR	FR-obs-1-P2C12*		
				FR-obs-2-D223	HM022852	KF286350
		*C. scoticus*	FR	FR-sco1-P7C5	HM022875	KF286351
				FR-sco2 P6C25	HM022857	KF286352
*Beltranmyia*		*C. circumscriptus*	TU	TU-cir2-B1Cf31*	KJ729971	KF286353
		*C. impunctatus*	FR	FR-del2-D91	KY707808	KF286355
				FR-del3-D94*		
*Culicoides*		*C. lupicaris*	FR	FR-lup1-P5C34	KY707776	KF286354
		*C. newsteadi*	TU	TU-new1-S3CM42	KKJ729989	KF286356
				TU-new2-S6Cf51	KJ729990	KF286357
		*C. punctatus*	FR	FR-pun1-D327	KY707806	KF286358
				FR-pun2-D242*		
				FR-pun3-D250*		
*Haematomyidium*		*C. limonensis*	EC	EC-para2-D460	KY707809	KF286360
		*C. paraensis*	EC	EC-para1-D423		KF286359
*Hoffmania*	*Guttatus group*	*C. diabolicus*	EC	EC-bat1-D451	KY707783	KF286361
				EC-bat2-D453	KY707787	KF286362
		*C. guttatus*	EC	EC-gu1-D481	KY707785	KF286363
	Hylas group	*C. hylas*	EC	EC-hyl1-D449		KF286364
		*C. pseudoheliconiae*	EC	EC-hyl2-D505	KY707784	KF286365
*Meijerehelea*		*C. distinctipennis*	GA	GA-leu1 D555	KY707792	KF286366
*Monoculicoides*		*C. nubeculosus*	FR	FR-nub-D179	KF178273	KF286367
		*C. parroti*	FR	FR-par-D27	KF178276	KF286368
		*C. puncticollis*	TU	TU-pco1-B7CM49	KJ730002	KF286369
				TU-pco2-B7Cf60*	KJ29998	KJ730024
*Oecacta*		*C. cataneii/*	TU	TU-cag1-B2CM132	KJ729968	KF286388
		*C. gejgelensis*		TU-cag2-B2Cf34	KJ729967	KF286389
		*C. festivipennis*	FR	FR-fes2-D66	KY707777	KF286377
				FR-fes3-D103*		
		*C. jumineri*	TU	TU-jum1-S3Cf124	KJ729979	KF286383
				TU-jum2-S3CM1*	KJ729980	KF286384
				TU-jum3-S3CM71	KJ729982	KF286384
		*C. sahariensis*	TU	TU-sah2-B28Cf6*	KJ30004	KF286387
*Remmia*		*C. enderleini*	MA	MA-scg2-D378*	KF186429	KF286379
				MA-scg3-D363		
		*C. kingi*	TU	TU-kin1-S3Cf167	KJ729985	KF286338
		*C. nevilli*	MA	MA-scg1-D357	KF186428	KF286378
*Trithecoides*		*C. fulvithorax/C. ochrothorax*	GA	GA-fuo1-D437	KY707793	KF286371
*Wirthomyia*		*C. segnis*	FR	FR-seg1-D113*	KY707778	KF286372
				FR-seg2-D108		
*Unplaced 1*	*Carpenteri group*	*C. belemensis*	EC	EC-bel1-D477	KY707786	KF286373
*Unplaced 2*	Fluvialis group	*C. castillae*	EC	EC-cast1-D474		KF286374
				EC-cast2-D472*		
		*C. tetrathyris* like	EC	EC-fug3-D470	KY707788	KF286375
		*C. tetrathyris*	EC	EC-tetra1-D517		KF286370
*Unplaced 3*	*Leoni* group	*C. glabellus*	EC	EC-leg1-D528	KY707789	KF286376
*Unplaced 4*	Milnei group	*C. moreli*	MA	MA-mor1-D420	KY707804	KF286382
		*C. zuluensis*	MA	MA-mig1-D388	KY707802	KF286380
				MA-mig2-D365	KY707803	KF286381
*Unplaced 5*		*C. paolae*	TU	TU-pao2-S5CM1	KJ729992	KF286385
				TU-pao3-S3Cf66	KJ729991	KF286386

EC: Ecuador; FR: France; GA: Gabon; MA: Madagascar; TU: Tunisia. D1D2 rDNA sequences (*specimens having identical sequences) and COI sequences).

Specimen identification was performed after mounting the head, wings and spermathecae on microscope slides, leaving the thorax and legs for subsequent DNA extraction [2]. Consequently, we were unable to identify *C. fulvithorax* and *C. ochrothorax* without their thorax that includes their discriminant character. Moreover, the accurate identification of females of some closely related specimens, such as *C. cataneii* and *C. gejgelensis,* was not possible [36]. Two specimens from Gabon, belonging to subgenus *Avaritia,* present new morphological characters compared with currently known species; hereafter we will refer to these specimens as *Culicoides sp*. At least two specimens of each species were sequenced, except for 13 species from which only one specimen was available (Table 1; Figs. 1–4).

A total of 68 specimens belonging to 42 species were analysed: 34 species belonging to the subgenera *Anilomyia, Avaritia, Beltranmyia, Culicoides, Haematomyidium, Hoffmania, Meijerehelea, Monoculicoides, Oecacta, Remmia*, *Trithecoides* and *Wirthomyia*, and 8 species belonging to unclassified groups [11]. Species distribution included: (i) Ecuadorian specimens (12 species) assigned to subgenera *Anilomyia, Haematomyidium* and *Hoffmania* and the unclassified groups Carpenteri group, Fluvialis group and Leoni group; (ii) French specimens (11 species) assigned to subgenera *Avaritia, Culicoides, Monoculicoides, Oecacta* and *Wirthomyia*; (iii) four Gabonese specimens assigned to subgenera: *Avaritia, Meijerehelea* and *Trithecoides*; (iv) Malagasy specimens (7 species) assigned to subgenera *Avaritia, Remmia* and to the Milnei group and (v) Tunisian specimens (8 species) assigned to six subgenera (*Avaritia, Beltranmyia, Culicoides, Monoculicoides, Oecacta, Remmia)* and 1 species was *C. paolae (incertae sedis)*.

### DNA extraction and PCR amplification

DNA was extracted from individual *Culicoides* using the QIAmp DNA Mini Kit (Qiagen GmbH, Hilden, Germany), following the manufacturer’s instructions. Polymerase chain reactions (PCRs) for D1-D2 and cytochrome oxidase genes were performed in a 50 μL volume using 5 μL of DNA solution and 50 pmol of primers C′1 (5′-ACCCGCTGAATTTAAGCAT-3′) and D2 (5′-TCCGTGTTTCAAGACGGG-3′) for D1-D2 [18] and C1J1718 (5′-GGAGGATTTGGAAATTGATTAGT-3′), C1N2191 (5′-CAGGTAAAATTAAAATATAAACTTCTGG-3′) or LepF (5′-ATTCAACCAATCATA AAGATA TTGG-3′) and LepR (5′-TAAACTTCTGGATGTCCAAAAAATCA-3′) for COI [2, 57].

Amplification conditions for D1-D2 were: initial denaturation step at 94 °C for 3 min followed by 35 cycles of denaturation at 94 °C for 30 s, annealing at 58 °C for 90 s and extension at 68 °C for 60 s followed by a final extension at 68 °C for 10 min. For COI amplification, conditions included: (1) initial denaturation step at 95 °C for 15 min, then 5 cycles at 95 °C for 40 s, 45 °C for 40 s, 72 °C for 1 min, were followed by 45 cycles at 95 °C for 40 s, 50 °C for 40 s, 72 °C for 1 min and a final extension step at 72 °C for 20 min for C1J1718/C1N2191, and (2) initial denaturation step at 94 °C for 3 min, 5 cycles of denaturation at 94 °C for 30 s, annealing at 45 °C for 90 s and extension at 68 °C for 60 s were followed by 35 cycles of denaturation at 94 °C for 30 s, annealing at 51 °C for 90 s, and extension at 68 °C for 60 s and a final extension at 68 °C for 10 min for LepF1/LepR. Amplicons were analysed by electrophoresis in 1.5% agarose gel stained with 0.1% ethidium bromide. All sequences obtained are available in GenBank (Table 1).

### Phylogenetic analyses

Most sequences of COI and D1-D2 genes were analysed separately and concatenated, except for four specimens (*Culicoides castillae, C. hylas, C. paraensis* and *C. tetrathyris*). The phylogenetic tree was constructed using both non-probabilistic (maximum parsimony, MP) and probabilistic approaches (Bayesian inference, BI), using *Atrichopogon sp*. and *Forcipomyia sp.* as outgroups [7].

Maximum parsimony analysis was carried out with PAUP* 4.0b10 [59] by selecting the heuristic search option with tree bisection reconnection branch swapping (TBR) and 1000 random sequence addition (RSA). All sites were equally weighed but a step matrix (ponderation TS/TV = 2) was applied. Sequences were edited and aligned manually using Se-Al [52]. The insertion of several interlocked gap zones was therefore necessary to align sequences. Sequence alignment was performed respecting the criteria defined by [6]: (1) to minimise the number of inferred mutations (number of steps); (2) to prefer substitution to insertion-deletion, and (3) to prefer transitions to transversions because they have a higher probability of occurrence.

A total of 423 bp and 613 bp were analysed for COI and D1-D2, respectively.

For the model-based approach, the best-fit model of nucleotide substitutions was computed with jModelTest v2.1.4 [16] using the Akaike Information Criterion (AIC). The Hasegawa, Kishino and Yano (HKY) +I+Γ model was indicated as the best-fit model for the mitochondrial COI gene. The general time reversible (GTR) +I+Γ model was indicated as the best-fit model for both D1-D2 and concatenated markers (rDNA marker D1D2 and COI). Bayesian analyses were carried out using MrBayes 3.1.2 [54] with 4,000,000 generations, 10,000 of the saved trees were discarded, and the 30,000 remaining were used to construct the resulting BI tree. The robustness of tree nodes was assessed by clade posterior probability values (CPP).

A first maximum parsimony analysis on COI sequences showed trees of 2786 steps with a consistency index (CI of 0.225) and a retention index (RI of 0.443). The codon position 3 in the COI gene was found to have saturated transition information as compared to position 1+2 (data not shown). Therefore, we decided to remove the codon position 3. A new analysis was performed with COI codon position 1+2 including 10,000,000 generations. As many as 25,000 of the saved trees were discarded. Both COI and D1-D2 sequences were analysed independently using the BI and MP approaches and a concatenated fragment using the BI approach.

## Results

A first run of amplification was carried out using the C1J1718/C1N2191 primers. Pseudogenes were amplified for six specimens from Ecuador (*C. castillae, C. hylas, C*. *pseudoheliconiae, C. guttatus, C. tetrathyris* and *C. diabolicus*) and one specimen from Gabon (*C. distinctipennis*). Consequently, the COI of these specimens was tentatively amplified and sequenced using the LepF1/LepR primers. Finally, the COI of all these specimens was obtained, except for *Culicoides castillae* and *C. tetrathyris.*

For the parsimony analysis, the sequences for COI with 127 variable characters, of which 97 were parsimony-informative were analysed. The most parsimonious trees obtained were 557 steps long. With D1D2 sequences, 203 variable characters were found, out of which 169 were parsimony-informative. The most parsimonious trees obtained were 639 steps long. The ribosomal gene had a much higher proportion of parsimony-informative sites than the mitochondrial gene (Table 2).

**Table 2.** Information about DNA sequences used in this study, in relation to one of the most parsimonious trees resulting from the combined analysis and mapped in a combined matrix.

DNA region	COI	D1-D2
Aligned matrix (bp)/number of characters included	423	661
Number of constant sites	352	456
Number of variable sites	127	203
Number of parsimoniously informative characters	97	169
Number of variable parsimony uninformative characters	30	34
Tree length (L)	557	639
CI	0.227	0.436
RI	0.361	0.689

Topologies of the trees obtained by MP and BI are presented in the Appendices. Our main findings were that: (i) the MP COI tree (data not shown) is unusable; (ii) the MP D1D2 tree (Appendix 1) shows that the subgenera *Monoculicoides, Culicoides, Haematomyidium* and *Remmia* were monophyletic, whereas the subgenera *Hoffmania* and *Avaritia* were paraphyletic; (iii) the BI COI tree (Appendix 2) shows that the subgenera *Avaritia*, *Culicoides, Hoffmania, Monoculicoides* and *Remmia* were monophyletic, whereas the subgenus *Oecacta* was paraphyletic, and (iv) the BI D1D2 tree (Appendix 3) shows that the subgenera *Culicoides, Hoffmania, Monoculicoides* and *Remmia* were monophyletic, whereas the subgenus *Oecacta* was paraphyletic.

**Figure 5.**
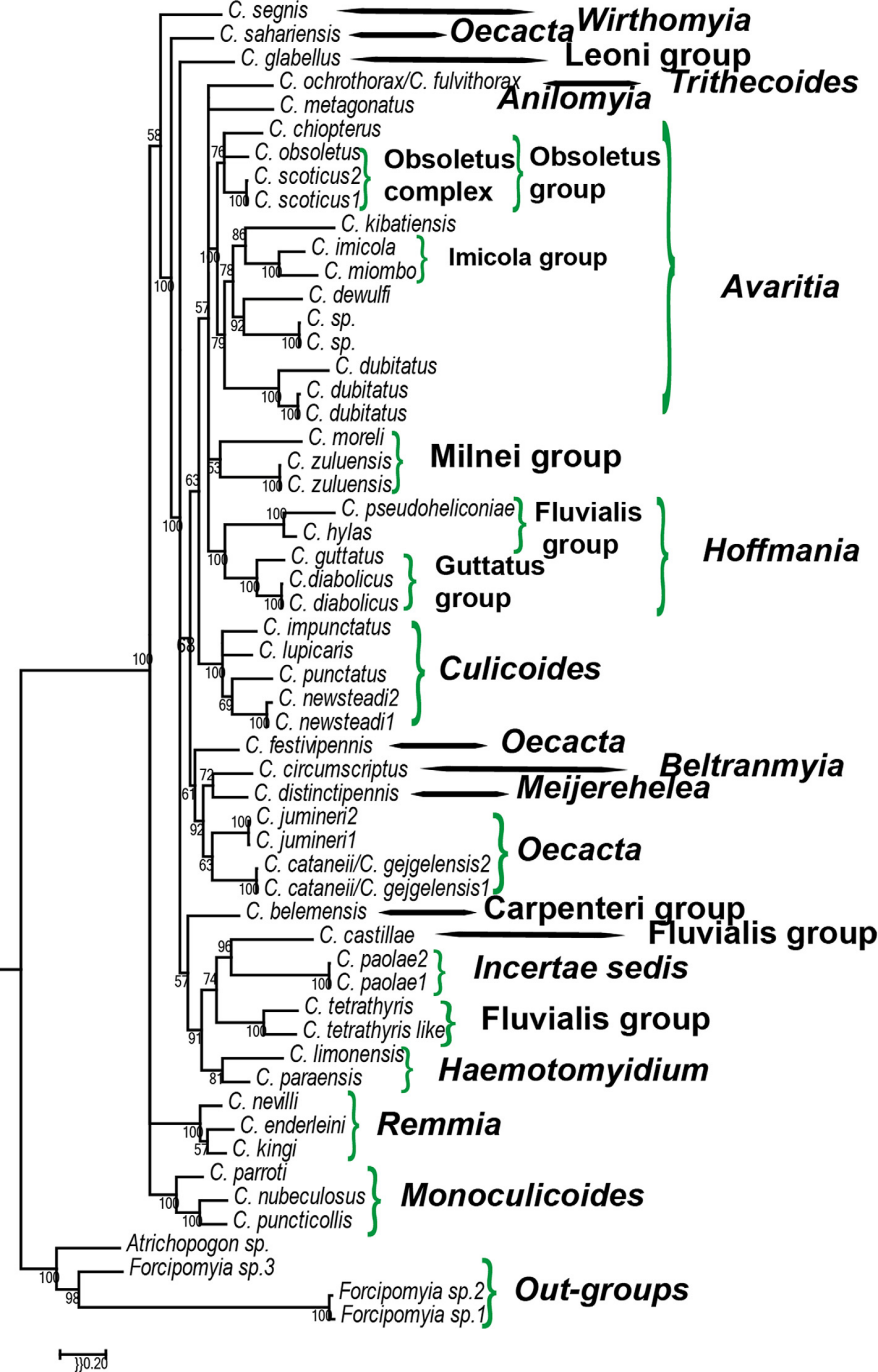
Bayesian tree resulting from the phylogenetic analysis of the concatenated dataset according to the best-fit partitioning strategy. Robustness of nodes is indicated by the posterior probability values (%).

Results of the concatenated Bayesian inference analysis of *Culicoides* relationships are shown in Figure 5 and presented below.

According to the combined data analysis, the genus *Culicoides* was clearly monophyletic.

The subgenus *Culicoides* was also monophyletic: Tunisian specimens of *C. newsteadi* were grouped in one clade (CPP = 100) with French specimens of *C. punctatus*, *C. lupicaris* and *C. impuctatus*.

The subgenus *Hoffmania* (species from Ecuador) displayed two clusters with *C. batesi* and *C. guttatus* as the sister group of the Hylas group. The tree shows that the subgenus *Hoffmania* was monophyletic (CPP = 100), and in the position of sister species of the Milnei group and the subgenera *Trithecoides, Anilomyia* and *Avaritia*.

The subgenus *Avaritia* was also monophyletic (CPP = 100). *C. chiopterus*, *C. obsoletus* and *C. scoticus* were grouped together as a clade, the sister group of all other members of the subgenus *Avaritia* (CPP = 78). *C. dewulfi* was shown to be closely related to the new *Culicoides* species from Gabon (CPP = 92). The analysis also revealed that the Imicola group was monophyletic. *C. imicola* s. st. is the sister species of *C. miombo* (CPP = 100) and *C. kibatiensis* is the sister species of them (CPP = 86).

The Schultzei group, which includes *C. enderleini*, *C. nevilli* and *C. kingi*, was monophyletic (CPP = 100).

The subgenus *Monoculicoides,* including *C. nubeculosus*, *C. parroti* and *C. puncticollis*, was monophyletic (CPP = 100).

*Culicoides distinctipennis* (subgenus *Meijerehela*) from Madagascar and *C. festivipennis* (subgenus *Oecacta*) from France exhibited similar wing patterns (white spots on the wing apex) but they were not grouped in our cladogram (CPP = 61).

The Milnei group, which includes *C. zuluensis* and *C. moreli*, was monophyletic (CPP = 53).

Bayesian analysis showed that the subgenus *Oecacta* was paraphyletic: *C. circumscriptus –* subgenus *Beltranmyia* and *Culicoides distinctipennis* – subgenus *Meijerehela* are included within the members of this subgenus, and *C. sahariensis* is separated from the other members of subgenus *Oecata*.

The monophyly of the Fluvialis group (CPP = 74) is discussed due to the inclusion in this clade of the *incertae sedis C. paolae*. The subgenus *Haematomyodium* is the sister group of the Fluvialis group (BI, CPP = 91).

## Discussion

The present study is, to our knowledge, the first systematic molecular analysis carried out on the genus *Culicoides* at a large taxonomic level, not focusing on closely related species (42 species belonging to 12 subgenera and unclassified groups collected in Afrotropical, Neotropical and Palaearctic areas). The data, based on COI and D1-D2 sequences, were subjected to a range of MP and BI analyses in order to explore the phylogenetic signal. To date, other molecular phylogenies, based mainly on COI sequences, were commonly restricted to a single subgenus or group located in Afrotropical or Palaearctic areas [29], except for one containing 37 Palaearctic species representing 10 subgenera [1]. Phylogenies studies based on ITS1 and ITS2 have also been reported on 25 French species [49] and 9 Italian species [26], respectively.

Pseudogenes are homologous sequences arising from currently or evolutionarily active genes that have lost their ability to function as a result of disrupted transcription or translation. They may contain stop codons, repetitive elements, have frame shifts and/or lack of transcription. However, they might retain gene-like features [65]. Pseudogenes have been identified in the mitochondrial genome of insects, also widely used in phylogenetic studies, with the risk of obtaining erroneous results during phylogenetic reconstruction [34]. To our knowledge, this is the first report of pseudogenes in *Culicoides*.

The subgenera *Anilomyia, Beltranmyia, Meijerehelea, Trithecoides, Wirthomyia* and some groups are only represented by a single species. Consequently, further studies are required to discuss their monophylies.

We demonstrate here the monophyly of the subgenus *Monoculicoides* that is in agreement with previous studies (ITS1: [49]*;* COI: [1, 55]).

Similarly, our results suggest that the subgenus *Culicoides* is also monophyletic, as previously reported by another study based on COI sequences [1], whereas studies based on ITS1 [32, 49], ITS2 [32] and COI [55] sequences suggested the paraphyly of this subgenus. Within this group, our findings suggest (according to molecular [33, 43, 48] and morphological studies [17, 38, 57]) the validity of *C. lupicaris*, whereas it is sometimes synonymised with *C. delta* [11].

Monophyly of the subgenus *Avaritia* is clearly supported by the present study, which corroborates previous results obtained from both morphological [46] and molecular data based on COI [15] and ITS1 [32, 49]. However, the paraphyly of the subgenus *Avaritia* has also been reported by analysis of COI [1, 50, 55] and ITS2 [32] sequences, taking into account the phylogenetic position of *C. dewulfi* outside this subgenus.

Most authors erroneously included *C. dewulfi* in the obsoletus complex or the obsoletus group [25, 41]. Our results clearly suggest that *C. dewulfi* does not belong to this group (Fig. 1) as previously emphasised by different molecular markers [25, 28, 43, 56]. Based on morphological, morphometrical and molecular data [2, 23, 28, 46, 47], *C. obsoletus* and *C. scoticus* are two closely related species belonging to the Obsoletus complex [5, 28, 60]. According to [42], the Imicola group in the Afrotropical region includes *C. imicola* and *C. miombo. C. kibatiensis* could present similar characters to *C. trifasciellus*, and subgroup *trifasciellus* is distinct from *Culicoides imicola* and the Imicola group [24]. *C. trifasciellus* belongs to the Orientalis group of the Afrotropical region [41] and its taxonomic position with *C. kibatiensis* is not resolved. Our results show that *C. imicola* is a cryptic species of *C. miombo*, *C. kibatiensis* being the sister species of the clade formed by these two species. Future studies are needed to carry out investigations at a subgeneric scale in order to determine the species status of the most important vector subgenera. Interestingly, the specimens collected in Gabon belong to the subgenus *Avaritia* and show unique morphological characters and nucleotide substitutions (COI). Therefore, a morphological description of this new species of *Culicoides* is in progress.

Our study validates the subgenus *Remmia* (= Schultzei group) as a valid subgenus, outside of the subgenus *Oecacta* [3, 11], and includes *C. kingi* [4, 14]. The status of the Schultzei group and its subgeneric affiliation has been disputed. It is sometimes included in the subgenus *Remmia* Glukhova [11, 19, 43], the latter considered by some authors as a junior synonym of the subgenus *Oecacta* (Poey) [14, 64] or sometimes unclassified [62]. Unlike many authors, we believe the subgenus *Oecacta* includes only the Furens group and/or perhaps the Schultzei group, if the first group is excluded from the latter as previously suggested [3, 14]. Future integrative taxonomy studies [27–29] should define this group with more precision, especially its relationships with the subgenus *Oecacta.*

*C. paolae* from Tunisia is included in the Fluvialis group constituted by New World species in our sampling (*C. castillae*, *C. fluvialis*, *C. tetrathyris* and *C. tetrathyris like*). The hypothesis that *Culicoides paolae* could be a synonym of the Central American species *C. jamaicensis* seems fair. Indeed, *C. paolae* and *C. jamaicensis* present huge morphological similarities [8, 44], thus raising the possibility that it was introduced into the Mediterranean Region at the time of Columbus, and was only discovered 500 years later and named *C. paolae* [44].

The subgenus *Hoffmania* is monophyletic with two clades (Hylas and Guttatus groups). To our knowledge, no recent data exist in the literature. This subgenus includes 82 extant species from South America and Asia. The wide geographic distribution warrants further studies of the subgenus *Hoffmania* using phylogenetic and integrative approaches at several scales.

The Milnei group is monophyletic. The species constituting this group have been described from the Afrotropical region [11] and transmit several pathogenic organisms, e.g. Akabane virus, BTV, Letsitele virus, unidentified virus isolates (Cul. 5/69*), Dipetalonema perstans* and *Dipetalonema streptocerca* [10]. To our knowledge, this group has been understudied and future integrative taxonomy studies [27–29] should more precisely define this group [24].

Palaearctic, Neotropical and Afrotropical *Culicoides* are mixed in our cladogram (Fig. 5) and there is no geographic clustering, indicating that the palaeobiogeography of the genus *Culicoides* does not follow the generalised tracks [37]. Consequently, *Culicoides* settled in different areas by wind [45], animal carriage [39] or by human activities [44]. For example, *C. imicola* specimens collected in Laos, Thailand, Vietnam and Reunion Island are of African origin [40]. In a *Culicoides* catalogue [11], 42 fossils were recorded from ambers from the Dominican Republic, the USA, Canada, Germany, Poland, the Baltic area and Russia, suggesting a Laurasian origin of the genus.

In conclusion, this study showed that the subgenera *Monoculicoides, Culicoides, Haematomyidium, Hoffmania* and *Avaritia* (including the main vectors of bluetongue disease) are monophyletic, whereas the subgenus *Oecacta* is paraphyletic. As proposed by Harrup et al. [29], a cladistic reinterpretation of the subgeneric classification of *Culicoides*, and species delimitations, should represent *Culicoides* taxonomy. Integrative taxonomy (including morphological, mitochondrial and other markers) and modern morphometric analysis (based on wing characteristics including type specimens) can help taxonomists as suggested by Hadj-Henni et al.

## Figures and Tables

**Appendix 1.**
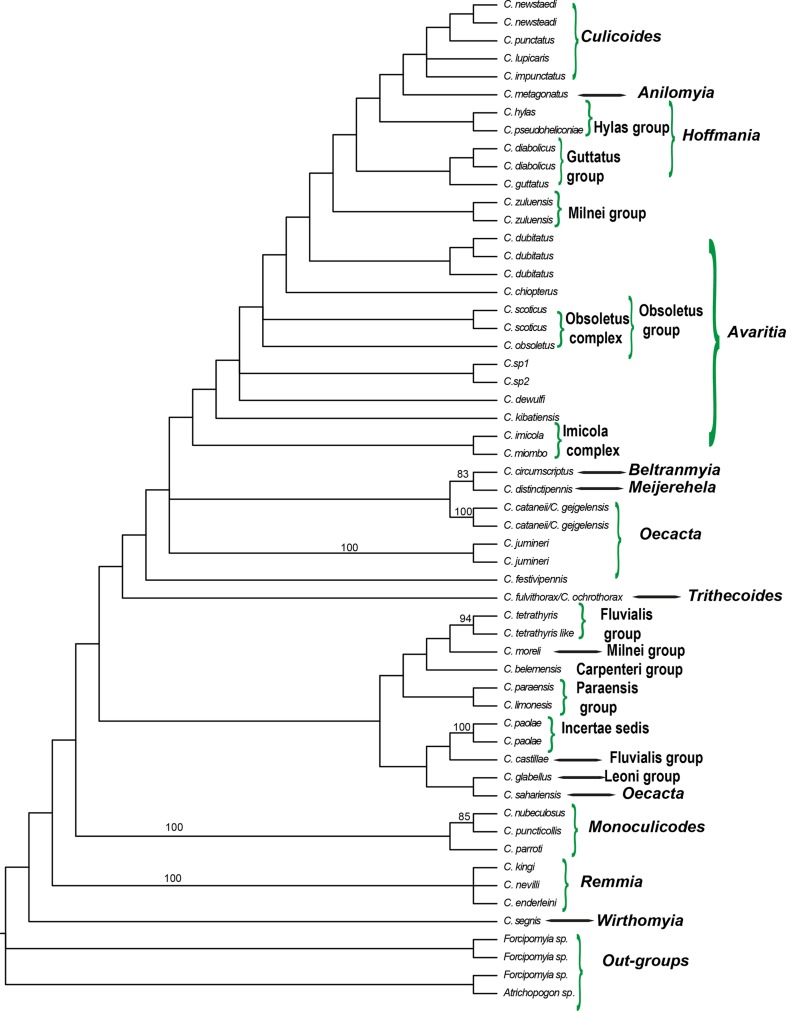
Parsimonious tree based on D1-D2 28S rDNA nucleotide sequences. Majority-rule 50% consensus: values between 50% and 100% are indicated on the branches.

**Appendix 2.**
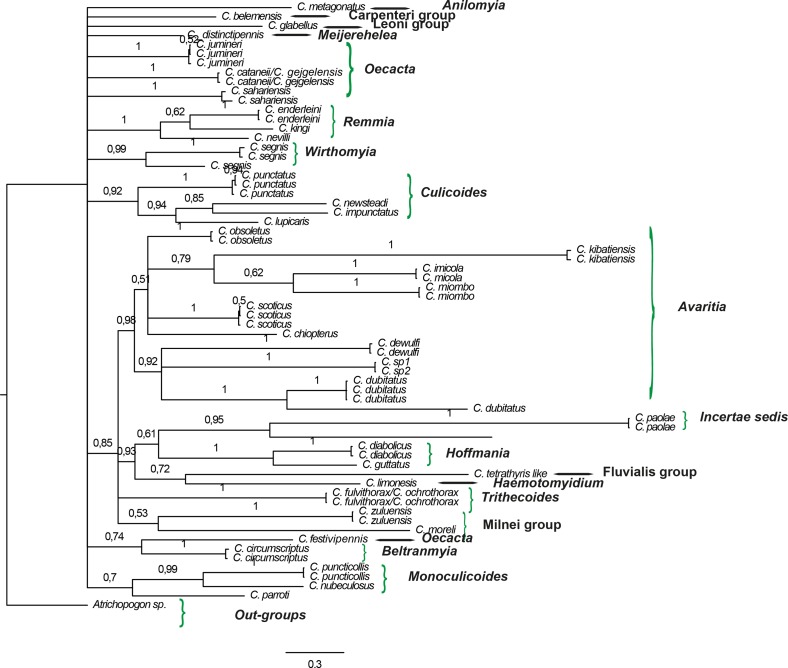
Bayesian tree resulting from the phylogenetic analysis of the COI mtDNA according to the best-fit partitioning strategy. Robustness of nodes was indicated by the posterior probability values.

**Appendix 3.**
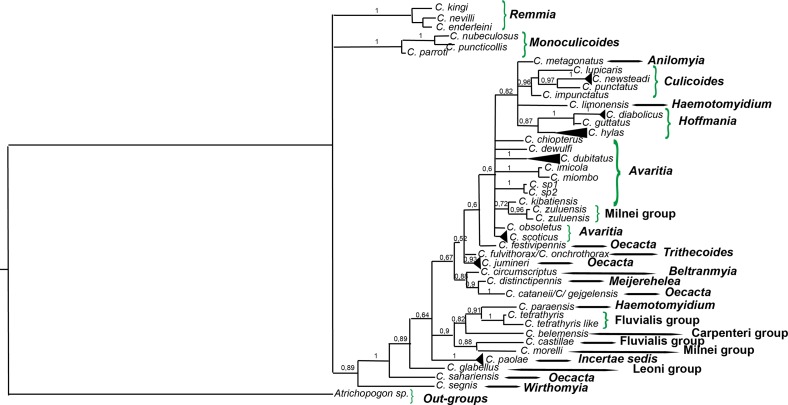
Bayesian tree resulting from the phylogenetic analysis of the D1D2 28S rDNA according to the best-fit partitioning strategy. Robustness of nodes was indicated by the posterior probability values.
